# Effect of slice thickness on brain magnetic resonance image texture analysis

**DOI:** 10.1186/1475-925X-9-60

**Published:** 2010-10-18

**Authors:** Sami J Savio, Lara CV Harrison, Tiina Luukkaala, Tomi Heinonen, Prasun Dastidar, Seppo Soimakallio, Hannu J Eskola

**Affiliations:** 1 Medical Imaging Centre, Tampere University Hospital, Biokatu 8, Tampere, FI-33521, Finland; 2 Department of Biomedical Engineering, Tampere University of Technology, Korkeakoulunkatu 3, Tampere, FI-33720, Finland; 3 Tampere University Medical School, University of Tampere, Medisiinarinkatu 3, Tampere, FI-33014, Finland; 4 Science Center, Pirkanmaa Hospital District, Biokatu 12, Tampere, FI-33521, Finland; 5 Tampere School of Public Health, University of Tampere, Medisiinarinkatu 3, Tampere, FI-33014, Finland; 6 Department of System Engineering and Architecture, Nokia Corporation, Visiokatu 1, Tampere, FI-33720, Finland

## Abstract

**Background:**

The accuracy of texture analysis in clinical evaluation of magnetic resonance images depends considerably on imaging arrangements and various image quality parameters. In this paper, we study the effect of slice thickness on brain tissue texture analysis using a statistical approach and classification of T1-weighted images of clinically confirmed multiple sclerosis patients.

**Methods:**

We averaged the intensities of three consecutive 1-mm slices to simulate 3-mm slices. Two hundred sixty-four texture parameters were calculated for both the original and the averaged slices. Wilcoxon's signed ranks test was used to find differences between the regions of interest representing white matter and multiple sclerosis plaques. Linear and nonlinear discriminant analyses were applied with several separate training and test sets to determine the actual classification accuracy.

**Results:**

Only moderate differences in distributions of the texture parameter value for 1-mm and simulated 3-mm-thick slices were found. Our study also showed that white matter areas are well separable from multiple sclerosis plaques even if the slice thickness differs between training and test sets.

**Conclusions:**

Three-millimeter-thick magnetic resonance image slices acquired with a 1.5 T clinical magnetic resonance scanner seem to be sufficient for texture analysis of multiple sclerosis plaques and white matter tissue.

## Background

Texture analysis (TA) is based on the examination of spatial patterns in image intensity. Many widely used texture analysis techniques exist in several fields of science, engineering and medical sciences. They have been successfully applied to several clinical applications, including multiple sclerosis (MS), brain injury and diseases that are otherwise difficult to identify at an early stage [1-3]. In neuroradiological imaging for clinical purposes, MS is the most common autoimmune disease of the central nervous system. It has a complex pathophysiology including inflammation, demyelination, axonal degeneration and neuronal loss. Diagnostic evaluation of MS is widely based on conventional magnetic resonance imaging (MRI) and the McDonald clinical diagnostic criteria [4,5]. The guidelines include evaluation of MS disease attacks, cerebrospinal fluid analysis and MRI. The MRI criteria includes three of the following 1) at least one active lesion seen on gadolium(Gd)-enhanced T1 images, or if there is no Gd-enhancing lesion at least nine T2 hyperintense lesions; 2) At least one infratentorial lesion; 3) At least one juxtacortical lesion; 4) At least three periventricular lesions. Spinal cord lesions can be considered as equivalent brain lesions. In McDonald criteria the lesion size has no relevance on the inclusion criteria for MS. Only the number of lesions is important.

As interest in texture analysis has grown due to its wide range of useful applications, a thorough understanding of the impact of different physical factors on it remains incomplete. For instance, sequence selection, magnetic field strength, voxel size, image resolution, patient movement, sequence-based noise and slice thickness are factors that complicate the interpretation of the acquired data. Some of these factors will be shortly discussed below. However, in our current study we concentrate on the effects of slice thickness on texture analysis and especially in brain MR images.

### Slice thickness

Herlidou-Meme et al. [6] have previously performed a multicenter texture analysis study in which three 1.5 T MRI units were used to acquire T1- and T2-weighted images with 2-mm, 4-mm and 6-mm slices for T1 and 2.5-mm, 5-mm and 7.5-mm slices for T2, respectively. Three classes of foam and gel test objects, ranging in size from 0.72-3.70 mm, were studied. According to their findings, the classification error was higher for small-scale objects, ranging from 10% for coarse foam to 40% for gel. They did not find the selection of slice thickness to be significant for classification accuracy, as the number of well-classified regions of interest (ROIs) seemed to be almost independent of slice thickness. However, their study included only a small set of objects.

Materka et al. [7] studied 1.5 T MR phantom images of foam-filled tubes with different fields of view (100 mm * 100 mm and 200 mm * 200 mm) and a constant number of image pixels (256 * 256) to find the effect of Gaussian noise and slice thickness (2 mm and 4 mm) on the Fisher coefficient F. Four texture classes were used with five samples in each. Only a few texture parameters with meaningful Fisher coefficient values were discovered. The number of useful parameters depended significantly on image normalization, as some texture parameters showed high correlation with the mean and variance. The Fisher coefficient decreased corresponding to the noise and slice thickness.

Guggenbuhl et al. [8] investigated the effect of slice thickness on texture parameters on computed tomography (CT) of calf bone images. They found that the selection of slice thickness used in the imaging had a significant influence on at least some run-length and co-occurrence parameters in the determination of bone microarchitecture. However, they did not perform any classification of textures in their study, and it remains unclear whether the classification accuracy of CT images would have been changed due to increased slice thicknesses.

### Spatial resolution

Jirak et al. [9] studied polystyrene spheres of varying diameters, ranging from 0.8 to 2.0 mm, as well as an agar solution, to investigate texture analysis procedures on MR images. They found feature extraction techniques, such as the Fisher coefficient and the probability of error with an average correlation coefficient (POE+ACC), to be very sensitive to deviations in inter-pixel relationships, noise and inhomogeneity. High resolution was often essential for the texture analysis of the analyzed materials. However, linear discriminant analysis (LDA) classification accuracy of objects with different spheres was over 90%, even in low-resolution images, thus suggesting that low-resolution images could be used for texture analysis. They also found that when high resolution was used the phantoms with small spheres and thus a more exact texture were easier to classify than those with larger ones, but with low resolution this was not true.

Mayerhoefer et al. [10] investigated the sensitivity of texture features to the variations in the number of acquisitions, repetition time (TR), time to echo (TE) and sampling bandwidth (SBW) in MR imaging using polystyrene spheres and agar gel phantoms. They found that texture features are increasingly sensitive to acquisition parameter variations as spatial resolution increases, but with a sufficient resolution, these variations did not have a considerable effect on the classification results in their study. As only relatively small test sets of spherical elements were used in these studies, it remains unclear whether the results can be fully generalized in the analyses of real acquisitions.

### Signal-to-noise ratio

Schad and Lundervold [11] showed that the best discriminating features of different textures depend on the signal-to-noise ratio (SNR), spatial resolution and voxel size, comparable to the natural properties of the imaged regions. A few texture parameters provide relatively constant results with all studied SNR levels and voxel sizes, but there is usually a connection between voxel size and the texture parameter values. For example, some Angular Second Moment values seem to increase as a function of increasing voxel size. The exact relationship varies between parameters and studied tissues. The stabilization of texture parameter values as a function of increasing SNR was observed in grey matter, but in white matter, the stabilization was less systematic.

### ROI selection

Harrison et al. [12] studied the sensitivity of texture analysis acquisition in MR images containing MS lesions at two different anatomical levels using Wilcoxon's signed ranks test. Several different tissues--white matter, normal appearing white matter, normal appearing grey matter, cerebrospinal fluid and MS plaques--were chosen for classification, and constant as well as manually drawn ROIs were used. According to the results, 96-100% of white matter (WM) or normal appearing white matter (NAWM) areas were classified correctly against MS plaques. No significant differences in the classification results for imaging sequences or anatomical levels were found in this study. The conclusion from this single center study was that texture analysis applied to MRI is a robust method when a fixed imaging sequence is used. However, they noted that the selection between a manually drawn and a rectangular ROI on an MS plaque depends on the specific application requirements and may have an impact on the accuracy of texture analysis.

### The purpose of our study

In this paper we provide a study of the effect of MRI slice thickness on detecting MS lesions by means of texture analysis of brain tissues and structures. There are usually numerous small lesions in the brain parenchyma of an MS patient, which may not be seen if the slice thickness is not small enough [13,14]. This means that the question of acquiring suitably thin slices and their effect on texture analysis plays an important role in the final diagnosis of the patient, especially as in earlier studies [7,8] it has been shown that many texture parameter values are dependable on the slice thickness.

If there are small lesions that are thin in axial direction, they may be mixed with the surrounding normal tissue present in thick slices. This underlines the importance of the slice thickness selection in the MR image acquisition. While many factors, such as the signal-to-noise ratio (SNR) and movement artifacts can influence the interpretation, we tried to make the analysis easier by averaging the intensities of three consecutive 1-mm slices to create a new slice, thus essentially expressing a 3-mm slice of the original subject.

The main aim of our study was to analyze the effect of slice thickness in the MRI examination for the detection of MS lesions using texture analysis. As far as we know, this effect has not been studied earlier by using real clinical material.

## Methods

We investigated the effect of slice thickness on the results from texture analysis of brain MR images acquired using a 1.5 T MRI device (Siemens, Avanto Syngo MR B15, Erlagen, Germany). We studied MR images of 23 clinically diagnosed MS patients (13 females and 10 males; mean age 42 ± SD 11; age range from 18 to 60 years). The participants' neuroradiological status based on MRI examinations fulfilled the McDonalds revised diagnostic criteria for MS [4,5]. None of the patients underwent steroid therapy 3-6 before MRI examinations.

The image analysis was focused at two anatomical levels, the corona radiata and the centrum semiovale, along with MS lesions, as well as basal ganglia. The present study forms a part of an ongoing study where 100 patients with CIS, RRMS, PPMS and SPMS are being longitudinally studied with yearly clinical and MRI control examinations.

Imaging was performed by the hospital's clinical imaging protocol for MS follow-up. This included standardized axial T1-weighted 3D magnetization prepared gradient echo sequence (MPR) (denoted by "T1") and the previous T1-weighted MPR sequence with the intra-venous contrast agent Gadoterate meglumine (Gd-DODA) (Dotarem^® ^10 ml) (denoted by "T1C"). The original slice thickness was 0.9 mm, which was interpreted as 1 mm for convenience. Other imaging parameters were field of view 230 mm*230 mm, TR 1160 ms, TE 4.24 ms, time for inversion (TI) 600 ms, echo train length 1, flip angle 15° and matrix size 512*512 pixels. To minimize imaging artifacts, a pre-scan normalization filter was used for the intensity inhomogeneity correction in the images. According to an experienced radiologist, the image sets were qualified as uniform, with good quality and free from motion artifacts.

**Figure 1:**
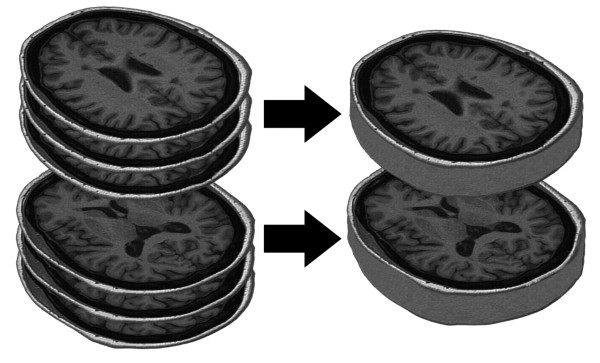
**Averaging of three sequential slices**. The intensities of three sequential slices at both imaging levels were averaged by Matlab to obtain simulated 3-mm slices. For each pixel in the simulated image, the corresponding pixel values of three consecutive 1-mm slices were added up and the sum divided by three, after which the result was rounded to the nearest integer.

The simulated 3-mm slices were created with Matlab (v. 7.7.0.471) [15] by intensity averaging of three sequential 1-mm slices, with the middle slice belonging to the studied set of 1-mm slices, see Figure 1. Each pixel intensity value in the simulated image was an average of the corresponding pixel intensity values of the above mentioned three consecutive 1 mm slices, rounded to the nearest integer.

**Figure 2:**
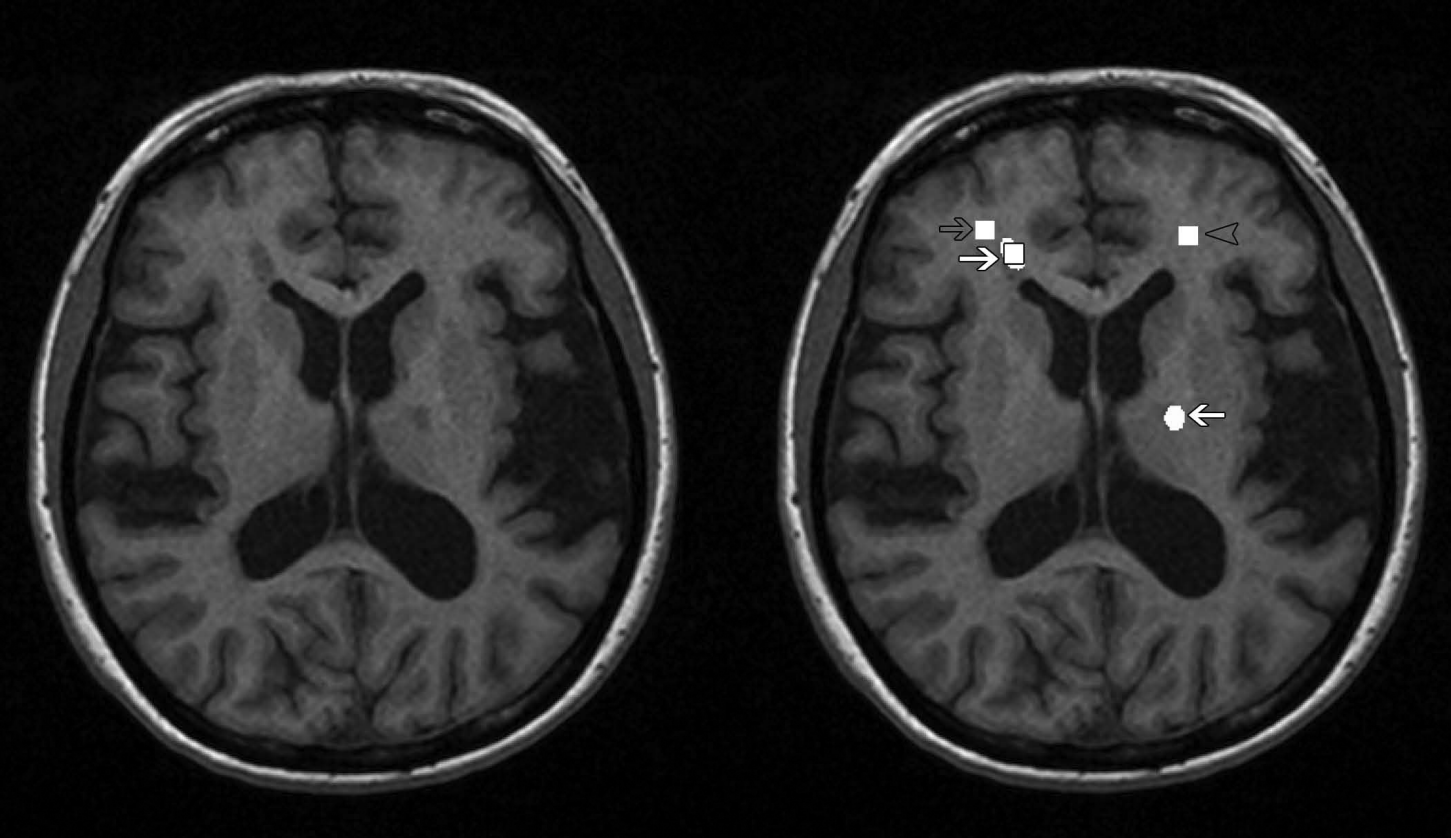
**Investigated ROIs**. On the left: An original T1 MPR image of an MS patient. On the right: ROIs drawn on the image. NAWM is indicated by a grey arrow and MS plaques by white arrows. (Both regular and hand drawn ROIs on an MS plaque are shown.) Normal white matter is indicated by a grey arrowhead.

Four ROIs were drawn in each original and simulated image: normal white matter (denoted by "WM"), normal appearing white matter adjacent to an MS plaque ("NAWM"), a manually drawn irregular ROI (size between 39 and 2477 pixels, medium size of 354 pixels) over an MS plaque ("MSi") and a constant rectangular ROI (size of 10*10 pixels) over an MS plaque ("MSr"). Rectangular ROI size was defined to fit on the average sized MS plaque. Image grey levels were normalized to limit image intensities to between [μ-3σ, μ+3σ] for each ROI, where μ is the mean grey level, and σ is the standard deviation. This normalization scheme was reported by Collewet et al. [16] to result in the best classification results in the case of MR imaging of soft cheeses and having no relationship between classification errors and MR acquisition protocols. An example of ROIs drawn is shown in Figure 2.

For each ROI, we calculated 264 texture parameter values by MaZda (v. 3.20) [17], a toolkit created by the European COST project [1]. These included 11 histogram parameters representing the gray level frequencies, 4 absolute gradient parameters showing neighboring pixel variation, 220 co-occurrence matrix parameters measuring probabilities of pixel pair occurrences, 20 run-length parameters to define frequencies of defined length and gray level, 4 wavelet and 5 autoregressive model-based parameters.

We made no assumptions for the data distribution and used Wilcoxon's signed ranks test to calculate a statistical measure for all texture parameters to find differences between ROIs. The resulting p-values that were smaller than 0.05 were considered as statistically significant, and the proportion of significant p-values was used as a separability measure.

We also tested the actual classification separability of each tissue pair by linear and nonlinear discriminant classifiers with Matlab [15]. If the training and test sets are not separate or they are small, there is a possibility that the classification results are too optimistic, or even that they are purely a coincidence in some cases. To avoid these risks and to take into account the fact that high correlation would probably exist between several texture parameters, we limited the amount of selected parameters for classifiers to three for each classification task by the Fisher coefficient. The Fisher coefficient was calculated in Matlab for all 264 texture parameters. The three parameters giving highest Fisher coefficient values for each classification task were included in the further classification procedures with linear and nonlinear discriminant classifiers. The Fisher coefficient F is defined as follows [7]:

$$F=\frac{D}{V},$$

where

*D *= between-classes variance and

*V *= within-classes variance.

When running linear and nonlinear discriminant classifiers, one hundred classifications with separate training and test sets were run for each comparison, and the median result was used as the measure for the classification accuracy. When the slice thicknesses for the training and test sets were different, we also made sure that the data from the same patient at the same physiological location did not belong to the training and test sets at the same time.

## Results

### Tissue separability based on Wilcoxon's signed ranks test

We calculated the texture parameter values for each combination of image sequences (T1 and T1C) and slice thicknesses (1 mm and simulated 3 mm). We also applied Wilcoxon's signed ranks test to these values. In the following analyses, each given separability measure is the percentage of statistically significant texture parameters when two ROIs are compared with a fixed slice thickness. In the following tables, "1 mm" refers to the original slices and "3 mm" to the simulated 3-mm slices. Table 1 shows the statistical separability between tissues based on Wilcoxon's signed ranks test.

**Table 1:** The percentage of statistically significant parameters

	1 mm		3 mm	
ROIs	T1	T1C	T1	T1C
WM vs. NAWM	12	1	11	11
WM vs. MSi	80	84	81	80
WM vs. MSr	76	74	78	84
NAWM vs. MSi	80	84	79	82
NAWM vs. MSr	75	78	77	80
MSi vs. MSr	66	73	71	73

The percentage of statistically significant parameters (p < 0.05) with slice thicknesses of 1 mm and 3 mm in Wilcoxon's signed ranks test for T1 and T1C (with contrast agent) images in comparison with white matter (WM), normal appearing white matter (NAWM), MS plaques with irregular ROI (MSi) and MS plaques with regular ROI (MSr). Each comparison includes 264 texture parameters.

Statistically significant differences were observed between tissues in the brain, both in the analyses of different slice thicknesses and in that of MRI sequences. Only the difference between WM and NAWM did not show high statistical significance.

We also calculated the Fisher coefficient for each texture parameter to select parameters for classification. Texture parameters that were chosen most often for the analysis by the Fisher coefficient value with sequence T1 were sigma and correlation at a distance of five pixels and a direction of 135 degrees (S(5,-5)Correlat), whereas the most used parameters for sequence T1C were sigma and sum variance at a distance of three pixels and a direction of 135 degrees (S(3,-3)SumVarnc). However, the chosen texture parameters depended on the compared ROIs as well as the slice thickness in the training set.

### Tissue classification results

The results of the linear discriminant analysis (LDA) between ROIs (excluding the MSi vs. MSr comparison) are shown in Table 2.

**Table 2:** The accuracy of LDA method for T1 and T1C

ROIs	1 mm -> 1 mm		1 mm -> 3 mm		3 mm -> 1 mm		3 mm -> 3 mm	
	T1	T1C	T1	T1C	T1	T1C	T1	T1C
WM vs. NAWM	60 [59, 61]	55 [53 56]	52 [50, 54]	50 [48, 53]	57 [55, 58]	55 [53, 56]	60 [59, 61]	62 [60, 63]
WM vs. MSi	92 [91, 95]	98 [97, 98]	93 [93, 94]	93 [92, 93]	92 [91, 95]	89 [89, 90]	96 [95, 96]	95 [94, 95]
WM vs. MSr	93 [93, 94]	93 [92, 94]	88 [88, 89]	91 [91, 93]	90 [90, 91]	84 [83, 85]	89 [88, 89]	92 [92, 93]
NAWM vs. MSi	91 [90, 92]	98 [97, 98]	92 [92, 93]	98 [98, 98]	95 [94, 96]	98 [98, 100]	95 [94, 96]	100 [98, 100]
NAWM vs. MSr	91 [90, 92]	90 [89, 91]	88 [86, 88]	90 [89, 90]	90 [90, 91]	91 [90, 92]	88 [86, 88]	94 [93, 94]

The accuracy of the LDA method in percentages for T1 and T1C (with contrast agent) images, in comparisons of white matter (WM), normal appearing white matter (NAWM), MS plaques with irregular ROI (MSi) and MS plaques with regular ROI (MSr). Each comparison includes three of the original texture parameters. Texture parameters have been chosen for LDA based on the Fisher coefficient. In the top row, "X -> Y" refers to the case in which the training set consists of slices with thickness X (1 mm/3 mm), whereas the test set comprises of slices with thickness Y (1 mm/3 mm). The 95% confidence interval for the median is shown in square brackets.

The classification results selected by linear discriminant analysis show that tissue classification can be performed reliably using both 1-mm and simulated 3-mm slices. This was applicable in the training set as well as in the test set.

The results of the nonlinear discriminant analysis (NDA) between ROIs (excluding MSi vs. MSr comparison) are shown in Table 3.

**Table 3:** The accuracy of NDA method for T1 and T1C

ROIs	1 mm -> 1 mm		1 mm -> 3 mm		3 mm -> 1 mm		3 mm -> 3 mm	
	T1	T1C	T1	T1C	T1	T1C	T1	T1C
WM vs. NAWM	59 [57, 60]	50 [48, 52]	54 [52, 56]	48 [46, 50]	54 [54, 55]	50 [50, 53]	57 [55, 58]	60 [59, 63]
WM vs. MSi	95 [94, 96]	96 [94, 97]	94 [93, 95]	92 [91, 93]	92 [91. 93]	88 [88, 89]	96 [96, 98]	93 [92, 94]
WM vs. MSr	92 [92, 93]	92 [91, 93]	85 [85, 86]	92 [91, 92]	90 [89, 90]	81 [80, 82]	88 [88, 89]	92 [91, 92]
NAWM vs. MSi	92 [91, 92]	98 [96, 98]	91 [90, 92]	97 [96, 98]	91 [90, 93]	98 [97, 98]	95 [93, 96]	98 [98, 100]
NAWM vs. MSr	90 [89, 90]	88 [88, 89]	86 [85, 88]	90 [89, 91]	89 [88, 90]	90 [89, 91]	86 [85, 86]	92 [91, 92]

Tissue classification with NDA. Results are given in percentages for T1 and T1C (with contrast agent) images, in comparisons of white matter (WM), normal appearing white matter (NAWM), MS plaques with irregular ROI (MSi) and MS plaques with regular ROI (MSr). Each comparison includes three texture parameters. Texture parameters have been chosen for NDA based on the Fisher coefficient. In the top row, "X -> Y" refers to the case in which the training set consists of slices with thickness X (1 mm/3 mm), whereas the test set comprises of slices with thickness Y (1 mm/3 mm). The 95% confidence interval for the median is shown in square brackets.

The performed nonlinear discriminant analyses show results similar to those presented in Table 2.

### The effect of slice thickness on texture parameter distributions

For each region of interest, we compared the texture parameter values for the original 1-mm slice and the simulated 3-mm slice in T1 and T1C images. The comparison was made by indicating the percentage of statistically significant p-values of 264 texture parameters. The results are shown in Table 4.

**Table 4:** Intra-tissue comparison between slice thicknesses

ROI	T1	T1C
WM	34	45
NAWM	44	42
MSi	57	67
MSr	51	59

Intra-tissue comparison between slice thicknesses. The percentage of statistically significant p-values (p < 0.05) when each region of interest (white matter (WM), normal appearing white matter (NAWM), MS plaques with irregular ROI (MSi) and MS plaques with regular ROI (MSr)) on a 1-mm slice is compared to the respective ROI on a 3-mm slice in T1 and T1C (with contrast agent) images. The comparison was made by indicating the percentage of statistically significant p-values of 264 texture parameters.

Intra-tissue comparisons with different slice thicknesses show statistical differences in texture parameter values. The texture parameter values with white matter show less statistically significant changes compared to that with MS plaques.

## Discussion

The aim of this study was to find direct applications for clinical analysis of TA in the field of MS. Our study included the evaluation of the effect of different slice thicknesses in MRI examination for detecting MS lesions using texture analysis. Our results show that only minor differences exist between different slice thicknesses. This can be clearly seen when the LDA and NDA classification results are analyzed.

### Two-dimensional vs. three-dimensional analysis

In this study two-dimensional analysis was performed even though three-dimensional texture analysis of brain images, applied by Mahmoud-Ghoneim et al. [18], was found to be a more exact method mathematically than its two-dimensional counterpart. However, the clinical evaluation remains mostly based on two-dimensional slices, and a fast two-dimensional texture analysis is easier to combine with this procedure. Furthermore, the slice thickness is normally much larger than the spatial resolution in each plane, and thus, non-cubical voxels are produced. For the clinician, it is also easier, faster and more reliable to draw ROIs two-dimensionally. In some cases only single slices are available, thus making three-dimensional analysis impossible.

### Data acquisition

In a sense it would have been more practical to acquire original 3 mm slices and compare their texture to the 1 mm ones. Our choice was a limited one regarding this because the MRI examinations of the MS patients studied here were already performed earlier using 1 mm thick slices, making our examination a retrospective one.

We synthesized thicker slices by averaging consecutive original slices. Both slice types were then studied to reveal the effect of slice thickness variation on texture analysis. Clearly, not all tissue properties are preserved in the averaging process and the real partial volume effect might have been a bit different. However, a clear advantage of this simple method is that even though the slice thickness was changed, the other basic parameters were left unchanged. The differences can thus be interpreted as the effect of thicker slices.

### Axial resolution

Brain tissue is not axially symmetrical, which is challenging for slice averaging. If several bones, muscles, or internal organs were to be studied, the differences between consecutive slices would probably be smaller. As Guggenbuhl et al. have noted [8], texture parameter values change when the slice thickness is altered. However, the influence of slice thickness on classification results seems to be relatively small in texture analysis of MS. The axial resolution of MR images decreases and SNR increases as the slices becomes thicker. These two factors have opposite effects on the accuracy of texture analysis, and their exact impact is case dependent. In the following subsections, we discuss the separability of tissues when two slice thicknesses are used.

### Statistical comparison of texture parameter values between ROIs

Based on our results, WM and NAWM seem to have the smallest mutual separability. This is true for both imaging sequences as well as for both slice thicknesses. A low value of separability was expected, as according to earlier studies [12,19], WM and NAWM are relatively hard to distinguish from one another with the clinically suitable 1.5 T magnetic field strength. Statistically significant p-values were found in only 11-12% of cases with T1 and in 1-11% with T1C (Table 1). Some of these values may even partly arise from the statistical significance level of 0.05 we have used.

When sequence T1 is considered, the ROIs that differ the most in statistical terms are WM compared to MSi (80-81% of p-values were statistically significant), NAWM to MSi (79-80%), WM to MSr (76-78%) and NAWM to MSr (75-77%). Even the ROI comparison of MSi to MSr (66-71%) provides a high proportion of texture parameters with statistically significant, low p-values. With sequence T1C, NAWM and MSi (82-84%) as well as WM and MSi (80-84%) are now the easiest ROIs to separate, followed by WM and MSr (74-84%) and NAWM and MSr (78-80%). In addition, the ROIs MSi and MSr are quite distinct from one another (73%) (Table 1).

When manually drawn ROIs are compared to the fixed size rectangular ROI on MS plaques it is important to note that manually drawn ROIs do not overlap with the neighboring tissues, whereas differences in lesion sizes between individuals lead to a variable amount of partial volume pixels in the standard-sized ROI boxes. Harrison et al. [12] expected this matter to have some impact on the accuracy of texture analysis. However, they state that standardized ROIs have several other advantages, including increased tolerance for slice selection and reproducibility.

We may also postulate that the average separability measure does not greatly depend on the selected imaging sequence or the slice thickness used, and the order of the separability values seems to be quite natural as well. White matter areas are very different compared to MS plaques. The separability of MS plaques from both WM and NAWM is high.

These findings are positive and suggest that many possible parameters are effective for the MS texture analysis and are independent of the slice thicknesses (1 mm or 3 mm) used in this study. In addition, in the study reported by Herlidou-Meme et al. [6], the classification results for foam were obtained almost independently of the slice thickness. It should be kept in mind, however, that in the mentioned study, the test set was relatively small.

When we compared WM and NAWM, the tissues were generally very difficult to separate; it also seems that the use of Gadoterate meglumine as a contrast agent does not have a significant influence on the texture classification accuracy.

### Tissue classification

We used linear discriminant analysis to test the real effect of the separability deduced by Wilcoxon's signed ranks test. The classification results show that MS and WM areas are well separable in terms of LDA classification. With some exceptions, WM and MS plaques were classified with at least 90% accuracy, as evident in Table 2. The classification with simulated 3-mm slices is almost as accurate as with 1-mm slices. The effect of sequence selection is rather small.

The results remain almost the same even if the discrimination decision was created based on the training set consisting of 1-mm slices and applied to simulated 3-mm slices in the test set, or vice versa. The order of classification accuracy by this method is also nearly the same as that suggested by Wilcoxon's signed ranks test; NAWM vs. MSi (91-100%), WM vs. MSi (89-98%), NAWM vs. MSr (88-94%) and WM vs. MSr (84-93%); therefore, the regular ROIs are harder to classify than the irregular ones.

The difference between 1-mm and simulated 3-mm slices in the linear classification of MS plaques and white matter thus seems rather small. One can also determine that the classification accuracy between NAWM and WM is poor, as could be expected from the separability results shown in Table 1.

We also tested NDA on the same ROIs and found that the classification results were nearly the same as in the case of LDA. From the results shown in Table 3, we see that, as in the case of LDA, the classification accuracy of NDA is also 90% or more in most cases, where WM or NAWM are classified against MS ROIs, and the order of the classification accuracy remains the same as that of LDA: NAWM vs. MSi (91-98%), WM vs. MSi (88-96%), NAWM vs. MSr (86-92%) and WM vs. MSr (81-92%). The classification accuracy between NAWM and WM is also poor with NDA.

### Comparison of texture parameter values between slice thicknesses

The selection of slice thickness seems to have a greater effect on the texture analysis of MS plaques than in the white matter areas. The results in Table 4 show the average statistical differences between texture parameter values calculated for 1-mm and simulated 3-mm slices. For both sequences T1 and T1C, the amount of statistically significant p-values is close to 50%, averaging 46% for T1 and 54% for T1C, when calculated over all four ROIs studied.

This separability measure ranges from 34% (WM) to 57% (a manually drawn irregular ROI over an MS plaque) when sequence T1 is considered, and from 42% (NAWM) to 67% (a manually drawn irregular ROI over an MS plaque) when T1C is examined. Textural differences in WM areas between 1-mm and simulated 3-mm slices seem to be decreased compared to those of MS plaques. These differences do not seem to affect classification results, as evident from the actual classification results shown in Tables 2 and 3. In general, there seems to be some statistical difference between 1-mm and 3-mm texture parameter distributions. The same effect in bone CT imaging was reported earlier by Guggenbuhl et al. [8]

### General findings

The interpretation of our findings is that the results from texture analysis vary slightly if the slice thickness is increased from 1-mm to 3-mm. As indicated by Schad and Lundervold [11], for example, textures also depend on spatial resolution and voxel size, and thus, it is important to investigate in which circumstances the texture can be identified. Changes in the texture due to the increased slice thickness are usually still small enough to enable adequate texture classification between MS plaques and white matter areas when suitable texture parameters are chosen. However, depending on the exact application, separate texture parameter sets for 1-mm and 3-mm slices may have to be used to enable sufficiently accurate classification. Although our findings are aligned with earlier studies, further clarification is still needed to understand the dependence of texture classification accuracy on slice thickness.

## Conclusions

In this study we found that replacing 1-mm slices with 3-mm-thick slices does not remove textures which would prevent a clinician from detecting the presence of lesions visible on 1-mm-thick slices. It is clear that very thin slices would produce insufficient SNR for reliable texture analysis, whereas very thick slices lack the texture details. Seemingly, the range from 1 mm to 3 mm does not correspond to either of these cases. To apply texture analysis in the quantification of clinical images, further studies are needed to show the effect of slice thickness as well as other parameters, such as movement artifacts and noise, on its performance.

## Competing interests

The authors declare that they have no competing interests.

## Authors' contributions

SJS participated in the design of the study, carried out the statistical image analysis and drafted the manuscript. LCVH designed the image analysis and participated in the statistical analysis and interpretation of the results. TL participated in the design of the study and helped to perform the statistical analysis. TH participated in the study design and coordination. PD participated in design and coordination as well as result interpretation. SS participated in the design and coordination of the study. HJE participated in the design, coordination and result interpretation. All authors participated in drafting the manuscript and read and approved the final manuscript.
